# 19. Delivery of Flu Immunizations to Healthcare Workers During COVID-19 Pandemic Using a Multidisciplinary Flu Team Approach in a Tertiary Center in the Middle East

**DOI:** 10.1093/ofid/ofab466.221

**Published:** 2021-12-04

**Authors:** Lyssette Cardona, Shafii Mohammed, Samah Nour, Mu’ed Alkhalaileh

**Affiliations:** 1 Cleveland Clinic Florida, Stuart, Florida; 2 Cleveland Clinic Abu Dhabi, Abu Dhabi, Abu Dhabi, United Arab Emirates

## Abstract

**Background:**

Compliance with influenza immunization in HCW remains a global challenge, uptake in the Middle East has been reported at 24.7% due to limited access and awareness (1). We aim to report a successful campaign by establishing a multidisciplinary Flu Team during ongoing COVID-19 pandemic.

**Methods:**

A multidisciplinary Flu team taskforce was assembled representing all stakeholders to include: Occupational Health, Nursing, Operations, Infomatics, Pharmacists and Administrative staff in July 2020. A pivot was made to switch location from previous year visits to an established vaccine center (ballroom) to a mobile campaign. From July to November 1st, the team met on a regular basis with 90 stakeholders to launch and monitor the ongoing immunizations. Electronic medical record (EPIC) tools such as One Click and Express Lane facilitated nursing check-in, documentation and immunization at one stop and eliminated previously used registration by other staff. EPIC Clarity feature facilitated reporting of compliance for managers and leadership. Ongoing education and awareness of immunization were ongoing through various platforms of communication such as huddles, phone screens, elevators, lounges, virtual grand rounds and corporate intranet communication and website videos.

**Results:**

Of the 3578 healthcare workers, 3,399 were immunized (95%) from September 2020 until the end of October 2020. There were 86 (2.4%) employees exempted during this period due to medical reasons or excused leaves ( e.g military, maternity), Figure 1. Compliance differed among functions, 95.86% physicians, 97.2% clinical and nursing, 92% academics, 94.96% finance, 91.15% human resources, 92.1% infomatics, 60% legal, 80.6% operations. Only 93 (2.6%) were non-compliant.

Employee Flu Immunization

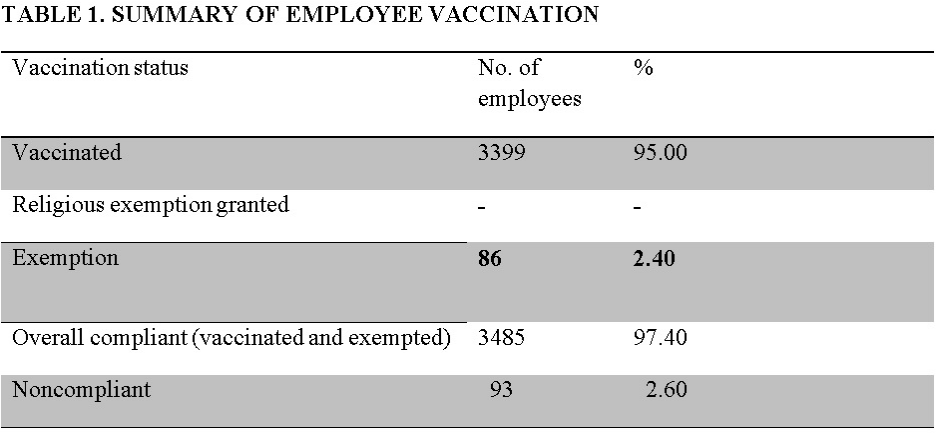

Hospital Overall Compliance in 2020

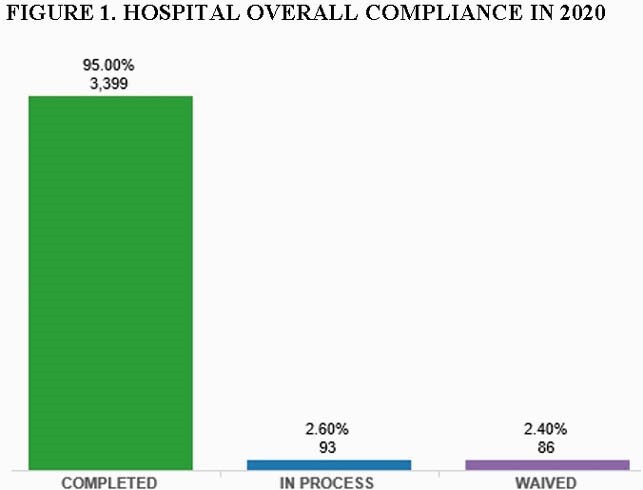

Compliance by Function in 2020

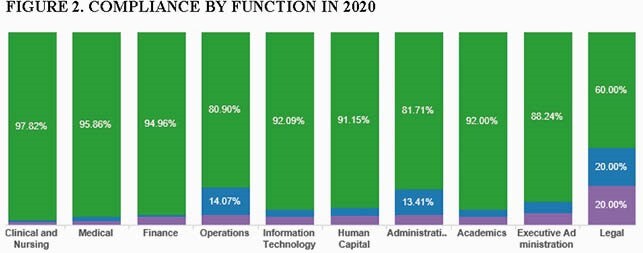

**Conclusion:**

Influenza illness adds an additional burden to the healthcare workforce during COVID-19. A multidisciplinary and collaborative team of teams approach delivered higher compliance for flu immunization than reported in the Middle East and enhanced by the use of state of the art technology. Convenience, educational awareness, free and safe access supported further the compliance with vaccination. To our knowledge, our 2020 flu campaign is the first successful experience reported in the Middle East during the current pandemic.

**Disclosures:**

**All Authors**: No reported disclosures

